# Transcriptome-Level Signatures in Gene Expression and Gene Expression Variability during Bacterial Adaptive Evolution

**DOI:** 10.1128/mSphere.00009-17

**Published:** 2017-02-15

**Authors:** Keesha E. Erickson, Peter B. Otoupal, Anushree Chatterjee

**Affiliations:** a Department of Chemical and Biological Engineering, University of Colorado Boulder, Boulder, Colorado, USA; b BioFrontiers Institute, University of Colorado Boulder, Boulder, Colorado, USA; University of Iowa

**Keywords:** adaptive resistance, CRISPR-Cas9, differential gene expression, gene expression variability, transcriptome

## Abstract

Antibiotic-resistant bacteria are an increasingly serious public health concern, as strains emerge that demonstrate resistance to almost all available treatments. One factor that contributes to the crisis is the adaptive ability of bacteria, which exhibit remarkable phenotypic and gene expression heterogeneity in order to gain a survival advantage in damaging environments. This high degree of variability in gene expression across biological populations makes it a challenging task to identify key regulators of bacterial adaptation. Here, we research the regulation of adaptive resistance by investigating transcriptome profiles of *Escherichia coli* upon adaptation to disparate toxins, including antibiotics and biofuels. We locate potential target genes via conventional gene expression analysis as well as using a new analysis technique examining differential gene expression variability. By investigating trends across the diverse adaptation conditions, we identify a focused set of genes with conserved behavior, including those involved in cell motility, metabolism, membrane structure, and transport, and several genes of unknown function. To validate the biological relevance of the observed changes, we synthetically perturb gene expression using clustered regularly interspaced short palindromic repeat (CRISPR)-dCas9. Manipulation of select genes in combination with antibiotic treatment promotes adaptive resistance as demonstrated by an increased degree of antibiotic tolerance and heterogeneity in MICs. We study the mechanisms by which identified genes influence adaptation and find that select differentially variable genes have the potential to impact metabolic rates, mutation rates, and motility. Overall, this work provides evidence for a complex nongenetic response, encompassing shifts in gene expression and gene expression variability, which underlies adaptive resistance.

**IMPORTANCE** Even initially sensitive bacteria can rapidly thwart antibiotic treatment through stress response processes known as adaptive resistance. Adaptive resistance fosters transient tolerance increases and the emergence of mutations conferring heritable drug resistance. In order to extend the applicable lifetime of new antibiotics, we must seek to hinder the occurrence of bacterial adaptive resistance; however, the regulation of adaptation is difficult to identify due to immense heterogeneity emerging during evolution. This study specifically seeks to generate heterogeneity by adapting bacteria to different stresses and then examines gene expression trends across the disparate populations in order to pinpoint key genes and pathways associated with adaptive resistance. The targets identified here may eventually inform strategies for impeding adaptive resistance and prolonging the effectiveness of antibiotic treatment.

## Abstract (precis)

Even initially sensitive bacteria can rapidly thwart antibiotic treatment through stress response processes known as adaptive resistance. Adaptive resistance fosters transient tolerance increases and the emergence of mutations conferring heritable drug resistance. In order to extend the applicable lifetime of new antibiotics, we must seek to hinder the occurrence of bacterial adaptive resistance; however, the regulation of adaptation is difficult to identify due to immense heterogeneity emerging during evolution. This study specifically seeks to generate heterogeneity by adapting bacteria to different stresses and then examines gene expression trends across the disparate populations in order to pinpoint key genes and pathways associated with adaptive resistance. The targets identified here may eventually inform strategies for impeding adaptive resistance and prolonging the effectiveness of antibiotic treatment.

## INTRODUCTION

By 2050, drug-resistant pathogens are predicted to lead to 10 million fatalities per year—more lives than are currently taken by cancer, traffic accidents, or diabetes (1). In order to avert a return to a preantibiotic dark age, new antibiotics are desperately needed, but in order to fully combat drug resistance, we must also strive to extend the useful lifetime of antibiotic drugs. The most concerning of pathogens have already evolved resistance to “nearly all” available treatments (2) and will undoubtedly rapidly subvert new drugs unless new strategies that thwart the emergence of resistance are implemented. At the heart of the antibiotic resistance crisis is the intrinsic processes that bacteria employ to survive and evade antibiotic treatment; these processes, which occur “upstream” of heritable resistance gains, are referred to as mechanisms of adaptive resistance (3). Adaptive resistance is generally considered a nongenetic response that temporarily facilitates increased likelihood of survival, with any tolerance gains reversing quickly upon removal of the stress (4). Interfering with the regulation of adaptive resistance could be a significant avenue by which to reduce the rise of novel resistances, but distinguishing key players in adaptive resistance is a demanding proposition. The main challenge lies in the vast heterogeneity introduced during adaptation; heterogeneity is essential to drive evolution (5), but divergence on both the genetic (6, 7) (i.e., mutational) and the nongenetic (4, 8, 9) levels confounds efforts to decipher the regulation of adaptive resistance. While much research effort has been devoted to scrutinizing mutational trends during adaptation (7, 10–13), only more recently have studies emerged considering nongenetic contributions to resistance (4, 14). Here, our motivation is to characterize the nongenetic basis for adaptive resistance, with the goal of providing fundamental insight that can be potentially applied to combat the emergence of bacterial drug resistance.

**FIG 1:**
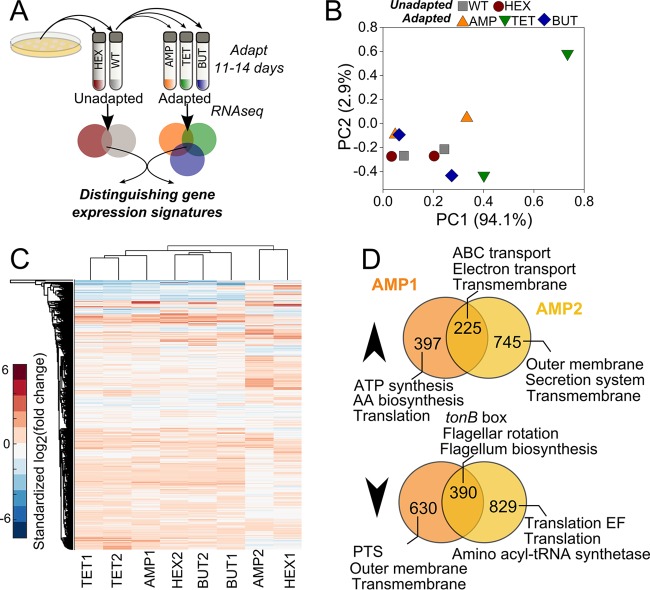
Heterogeneity in gene expression upon adaptation. (A) To obtain adapted and unadapted populations, individual wild-type (WT) *E. coli* K-12 MG1655 colonies were picked from plates and used to inoculate liquid cultures. Wild-type and* n-*hexane (HEX) samples were harvested after 1 day of growth in M9 minimal medium. Ampicillin (AMP), tetracycline (TET), and* n-*butanol (BUT) populations were collected after 11 to 14 days of adaptation. (B) Principal-component (PC) analysis of normalized transcript abundance (using FPKM) in the 10 populations (two populations per condition). (C) Heterogeneous gene expression patterns are observable across independent populations. Color indicates gene expression in indicated sample (*x* axis) with respect to duplicate wild-type populations. Values of log_2_(fold change) are standardized by sample such that the mean for each sample is 0 and the standard deviation is 1. Clustering is according to Euclidean distance. (D) Similarities and differences between ampicillin-adapted populations 1 and 2. Venn diagrams list the number of genes overexpressed ≥2-fold or underexpressed (indicated by up or down arrowheads, respectively) in each or both populations. The three most enriched gene ontologies are called out for each condition. AA, amino acid; EF, elongation factor; PTS, phosphotransferase.

To enable identification of general gene expression signatures associated with adaptive resistance, and not those related to a specific toxin, we specifically sought to generate diversity within and across adapted populations. To achieve this end, we adapted duplicate *Escherichia coli* K-12 MG1655 colonies to each of three toxins with dissimilar mechanisms of action: ampicillin (targeting cell wall synthesis), tetracycline (targeting translation), and *n*-butanol (a complex stress, impacting the membrane, metabolism, and respiration [15]). Adaptation was carried out for 11 to 14 days to approximate a standard antibiotic course (Fig. 1A; Table 1). Previous research has explored transcriptome-level responses in bacteria upon exposure to each of these stresses (15–17). It has been shown that gene expression signatures can be used to predict the specific mechanism of action for many antibiotics, including tetracyclines and penicillins (17); however, antibiotic exposure has been linked to a multitude of nonspecific responses, including stress response pathway activation, mutation rate changes, and membrane modifications (16, 18–20), which can confound efforts to find key regulators of adaptive resistance.

**TABLE 1:** Growth conditions for sample populations^a^

Sample	No. of days propagated	Medium condition at time of sampling(M9 minimal medium with 0.4% glucose and toxin as stated)
Unadapted		
WT1	1	NA
WT2	1	NA
HEX1	1	10.0% (vol/vol)* n-*hexane
HEX2	1	10.0% (vol/vol)* n-*hexane
Adapted		
AMP1	14	100 μg/ml ampicillin
AMP2	12	100 μg/ml ampicillin
TET1	14	4 μg/ml tetracycline
TET2	11	4 μg/ml tetracycline
BUT1	14	1% (vol/vol)* n-*butanol
BUT2	14	0.5% (vol/vol)* n-*butanol

a Abbreviations: NA, not applicable; WT, wild type; HEX, hexane; AMP, ampicillin; TET, tetracycline; BUT, *n*-butanol.

During the course of the adaptation period, the MIC for ampicillin-adapted populations increased 25-fold to reach 200 µg/ml of ampicillin, the MIC for tetracycline-adapted populations increased 8- to 16-fold to 8 µg/ml tetracycline, and *n*-butanol-adapted populations saw no net increase in tolerance, maintaining a MIC of either 1 or 2% (vol/vol) *n*-butanol. Unadapted samples were harvested from bacteria grown either in M9 minimal medium without selection pressure or in that medium with *n*-hexane, a compound to which *E. coli* is intrinsically resistant (21). To measure the gene expression patterns dominating in each population, we harvested RNA from unadapted and adapted populations and sequenced the resulting libraries on an Illumina HiSeq 2000 sequencer. We probed the transcriptome data with traditional gene expression analysis as well as an unconventional analysis using gene expression variability. Our results demonstrate the presence of immense transcriptome-level heterogeneity in gene expression even between replicates adapted to the same toxin and then filter that information to arrive at a focused set of 16 genes with conserved signatures in either expression (11 genes) or variability (5 genes) across diverse adapted bacterial populations. We investigate the upstream regulators of these genes to detect major networks controlling adaptive resistance and use clustered regularly interspaced short palindromic repeat (CRISPR) interference to perturb expression of target genes and measure impact on adaptation. In total, this work underlines the complexity of intrinsic bacterial adaptation mechanisms and presents key genes and pathways involved in general adaptive resistance.

## RESULTS AND DISCUSSION

### Extensive interpopulation gene expression heterogeneity upon adaptation.

We have previously examined gene expression heterogeneity in a select set of stress response genes in *E. coli* populations adapted to ampicillin, tetracycline, and *n*-butanol (22); here, we explore divergence at the transcriptome level. As a gauge of the degree of interpopulation gene expression heterogeneity, we performed principal-component analysis (PCA) on normalized transcript abundance within each population (as fragments per kilobase of transcript per million mapped reads [FPKM]) (Fig. 1B). PCA shows that the expression patterns do not group according to the stress condition. While the wild-type and* n-*hexane samples are relatively close in PCA space, the adapted populations are more widely distributed. This indicates that the overall gene expression profiles have been impacted as a result of the adaptation, but different populations have achieved unique solutions. Clustering according to the resulting relative gene expression levels [log_2_(fold change) from Cufflinks] gives additional insight into the heterogeneity in the adapted populations (Fig. 1C). Across all samples, on average most genes (56% ± 9%) had expression levels that were within 2-fold of that of the wild type. Underexpressed genes (at least a 2-fold decrease with respect to the wild type) represented 28% ± 4% of genes, while 16% ± 5% of genes were overexpressed (≥2-fold increase). Furthermore, the PCA (Fig. 1B) and clustering analysis (Fig. 1C) reveal that groupings are not fully explained by differences in optical density (OD) at the time of sampling. For instance, in PCA space WT1 (OD = 0.59) and HEX2 (OD = 0.64) are relatively close together but very far from TET2, which has an optical density of a similar magnitude (OD = 0.63). Likewise, in Fig. 1C BUT2 had an OD of 0.83 at the time of sampling and yet is clustered with HEX2 (OD = 0.64) rather than AMP2 (OD = 0.84). Therefore, we conclude that factors beyond differences in growth condition are contributing to the heterogeneity observed between populations.

Comparison particularly between populations adapted to the same selection pressure reveals substantial differences (Fig. 1C and D; see also Text S1 in the supplemental material). For example, across ampicillin populations, there were 397 and 745 genes with ≥2-fold-higher expression in populations 1 and 2, respectively; out of these, 225 were common between populations. The three most enriched functional classifications for the overexpressed set of genes in population 1, as obtained from DAVID v. 6.8 (23), include ATP synthesis (enriched 40-fold, *P* = 8.8e−21), amino acid biosynthesis (33-fold, *P* = 8.7e−9), and translation (22-fold, *P* = 1.8e−42). In population 2, outer membrane (enriched 87-fold, *P* = 4.1e−25), secretion system (41-fold, *P* = 5.7e−6), and transmembrane (4.7-fold, *P* = 1.9e−127) were the most enriched functions within overexpressed genes. Enriched functions for genes overexpressed in both populations included ATP-binding cassette (ABC) transporters (enriched 61-fold, *P* = 3.3e−21), electron transport (25-fold, *P* = 3.9e−6), and transmembrane (4.6-fold, *P* = 2.2e−27). Several transport and membrane-associated genes were overexpressed in both ampicillin populations, including *nikBC*, *tauC*, and *gltJ*. Differences lay in the particular set of genes impacted; for instance, the *emrA* and *acrB* multidrug efflux genes had higher expression in population 1 (no change in population 2), while alternate multidrug efflux genes (e.g., *mdtB*, *mdtL*, and *emrY*) were overexpressed in population 2 (no change in population 1). Other notable differences include higher expression of cellular division genes (*ftsQ*, *ftsB*, and *ftsL*) and NADH-quinone oxidoreductase genes (*nuoBC*, *nuoEF*, and *nuoGHIJKLMN*) in ampicillin population 1 versus higher expression of various transcriptional regulators (*lsrR*, *allS*, *cynR*, *envR*, and others) in population 2.

10.1128/mSphere.00009-17.1 TEXT S1  Detailed materials and methods as well as additional results and discussion. Download TEXT S1, PDF file, 0.4 MB. Copyright © 2017 Erickson et al. 2017 Erickson et al. This content is distributed under the terms of the Creative Commons Attribution 4.0 International license.

Differences were also present in underexpressed genes. In population 1 only, 630 genes were underexpressed; the three most enriched functions were phosphotransferase system (enriched 62-fold, *P* = 2.4e−8), outer membrane (43-fold, *P* = 2.3e−14), and transmembrane (4.3-fold, *P* = 1.3e−104). In population 2 only, the 829 underexpressed genes included many other transport and membrane proteins, as well as sensor proteins (e.g., *phoQ*, *dcuS*, and *rcsD*) and transcriptional regulators (e.g., *uvrY*, *narL*, and *baeR*). The most enriched functions among underexpressed genes in population 2 were all associated with protein synthesis: translation elongation factor (enriched 470-fold, *P* = 2.6e−15), translation (22-fold, *P* = 1.4e−66), and aminoacyl-tRNA synthetase (150-fold, *P* = 3.5e−19). Within the 390 genes underexpressed in both ampicillin populations, enriched functions were *tonB* box (470-fold, *P* = 2.6e−15), flagellar rotation (380-fold, *P* = 1.2e−14), and flagellum biosynthesis (190-fold, *P* = 1.9e−10). The *tonB* box is a binding sequence that is present in all TonB-dependent transporters, which have substrates including ferric siderophores, nickel chelates, vitamin B_12_, and carbohydrates (24).

We observed a multitude of differences between the duplicate tetracycline- and* n-*butanol-adapted populations as well. Across tetracycline populations, 172 genes had ≥2-fold overexpression in population 1 only (histidine biosynthesis was enriched 22.4-fold, *P* = 9.7e−1) and 235 genes had ≥2-fold overexpression in population 2 only (leucine biosynthesis enriched 13.3-fold, *P* = 2.7e−2). Across* n-*butanol populations, 156 and 282 genes had ≥2-fold overexpression in only population 1 or 2, respectively (biotin synthesis enriched 25.2-fold, *P* = 9.6e−1, in population 1, and colanic acid biosynthesis enriched 8.1-fold, *P* = 7.0e−8, in population 2). We have provided further discussion on the differences in gene expression in tetracycline- and* n-*butanol-adapted populations in Text S1 in the supplemental material.

### Transcriptome-level signatures based on differential gene expression.

**FIG 2:**
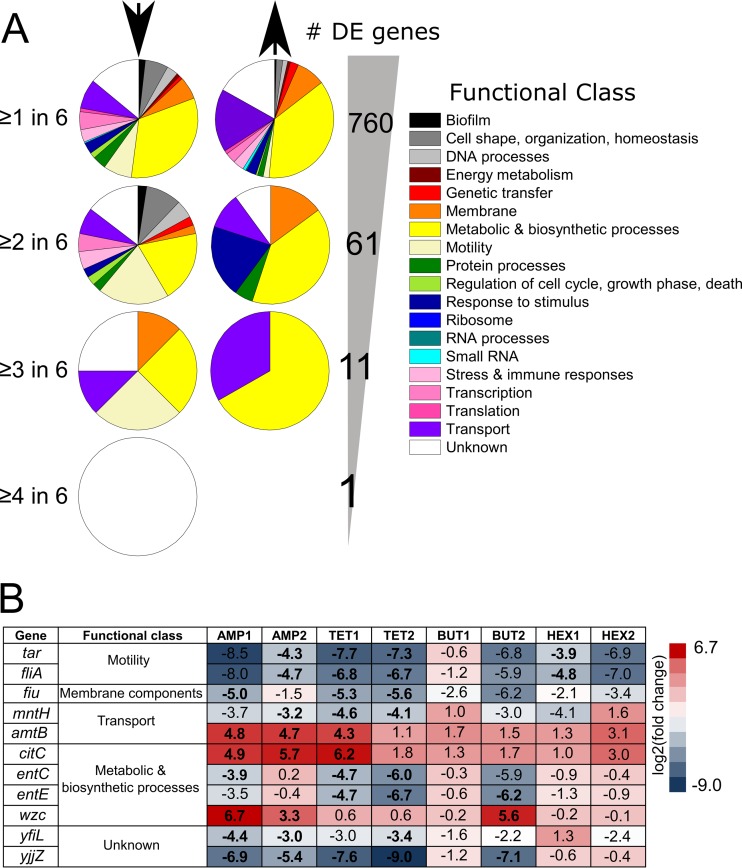
Intersections in differentially expressed (DE) genes across adapted populations. (A) Gene ontology distribution is shown for genes found to be differentially expressed or overexpressed (indicated by down or up arrows, respectively) in at least one, two, three, or four out of six adapted populations. The total number of genes DE at each level is shown to the right of the pie charts. (B) Summary of the 11 genes that were significantly DE in at least half of the adapted populations. Gene expression values are bold if the gene was significantly differentially expressed (*P* < 0.05, *q* < 0.3).

In light of the heterogeneity in gene expression, we calculated differential gene expression using partial replicates for each toxin and determined the expected variability in each adapted population according to the variance between the duplicate wild-type libraries (i.e., with a pooled dispersion metric). To attain a reduced set of genes most likely contributing to general adaptive resistance, we selected a false discovery rate of 30% to obtain the differentially expressed (DE) genes within each population and then filtered to locate intersections across populations (Fig. 2A). As expected, few genes were significantly DE in two populations exposed to the same toxin (10 genes shared between the two ampicillin populations, 24 genes between tetracycline populations, none between* n-*butanol populations, and 1 between* n-*hexane populations). Overall, a total of 760 unique genes were DE across all six adapted populations. Upon filtering to include only genes DE in at least two out of the six adapted populations, we were left with 61 genes with a variety of annotated roles (Fig. 2A). The top three classifications associated with underexpressed genes included metabolic and biosynthetic processes (20%), cell motility (17%), and genes with unknown function (15%). The top three classifications for overexpressed genes included metabolic and biosynthetic processes (35%), membrane components (20%), and response to stimulus (20%). Twenty genes encode enzymes present in the *E. coli* genome-scale metabolic network model iJO1366 (25), including *gatD*, *gmd*, *tnaA*, *hisG*, and *fes*, among others (Fig. S3). A complete list of DE genes is available in Data Set S1, along with a summary of overrepresented gene ontologies (23) in the entire set of DE genes.

10.1128/mSphere.00009-17.2 FIG S1  Growth curves for adapted and unadapted populations. Time is from 0 to 24 h for all. The natural log of the normalized optical density (OD at time *t* divided by the OD at time *t* = 0) is plotted for the strain and condition as indicated. The sampling points at which cultures were harvested for RNA sequencing are indicated by vertical lines. All strains were grown in M9 minimal medium with 0.4% glucose. For *E. coli* MG1655 in medium with no pressure, *n* = 8. For *E. coli* MG1655 in 10% (vol/vol)* n-*hexane, *n* = 9. For all others, *n* = 3. Error bars represent SD. Download FIG S1, TIF file, 0.1 MB. Copyright © 2017 Erickson et al. 2017 Erickson et al. This content is distributed under the terms of the Creative Commons Attribution 4.0 International license.

10.1128/mSphere.00009-17.3 FIG S2  Fold change for six genes with respect to wild type shown for measurements using RNA sequencing (DESeq and Cufflinks analysis) and qPCR, as indicated. A value of 1 indicates that gene expression is close to average wild-type expression levels. Fold change for qPCR is calculated as previously described (22), as the normalized relative quantity using the 2^−ΔΔ*Cq*^ method. Download FIG S2, TIF file, 0.7 MB. Copyright © 2017 Erickson et al. 2017 Erickson et al. This content is distributed under the terms of the Creative Commons Attribution 4.0 International license.

10.1128/mSphere.00009-17.4 FIG S3  (A) Genes present in the *E. coli* genome-scale metabolic model iJO1366 that were differentially expressed in at least 2 adapted samples, categorized by functional class. (B) Table listing the genes and their respective subsystems and the direction of the gene expression change. Download FIG S3, TIF file, 2.1 MB. Copyright © 2017 Erickson et al. 2017 Erickson et al. This content is distributed under the terms of the Creative Commons Attribution 4.0 International license.

10.1128/mSphere.00009-17.9 DATA SET S1  This file contains four tabs. (1) Differential expression. Contains information on differentially expressed genes. Table includes only those genes determined to be significantly DE via Cuffdiff (with *P* < 0.05 and *q* < 0.3). (2) Expression variability. Table includes 4,181 genes that pass FPKM filters. Genes were determined to be DV by examining changes in the CV of the FPKM between adapting and unadapting populations (*P* < 0.05 and *q* < 0.3). (3) Upstream regulation of target genes. Number in column titled “Level” indicates how many layers above which that gene impacts the 11 common DE or 5 DV genes. For instance, a number of 0 indicates that the gene is one of the target genes, 1 indicates that the gene directly regulates a downstream target gene, 2 indicates that the gene regulates a downstream gene that further regulates one of the target genes, etc. In all cases, the lowest level possible is recorded, though genes can regulate at multiple levels (e.g., *fliA* regulates *tar* and so could be a level 1, but since *fliA* is also a target gene, it is assigned a 0). (4) Enriched functional classes in differentially expressed or differentially variable genes. Enriched groups and genes in each were identified using the DAVID functional clustering tool with the highest classification stringency. Associated biology is manually summarized based on all gene ontology enrichment terms in each functional cluster. Download DATA SET S1, XLSX file, 1.1 MB. Copyright © 2017 Erickson et al. 2017 Erickson et al. This content is distributed under the terms of the Creative Commons Attribution 4.0 International license.

Filtering further, we identified 11 genes that were DE in at least three out of the six adapted populations (Fig. 2A and B), including nine genes representing functional classes of motility, membrane components, transport, and metabolic and biosynthetic processes and two of unknown function. The adapted populations in which these genes were DE and the degree of the expression change are shown in Fig. 2B. All nine genes of known function have previously been linked, either directly or indirectly, to various stress responses, providing corroboration that these genes participate in adaptive resistance. Among underexpressed genes, the transport gene *mntH*, the membrane protein gene *fiu*, and the enterobactin synthesis genes *entC* and *entE* are associated with iron scavenging. Previous literature shows that changes in regulation of iron metabolism instigate mutagenesis (26) in systems including *Mycobacterium tuberculosis* response to phage (27). The motility-associated genes *fliA* and *tar* were also underexpressed upon adaptation to ampicillin or tetracycline. Out of the entire set of 11 DE genes, these two motility genes were the only ones also found to be DE in either of the* n-*hexane populations (*n*-hexane population 1). Decreased expression of motility genes has been previously seen in similar adaptive evolution experiments (8) and has been hypothesized to be a generalized means of self-protection via energy conservation under particularly harsh conditions (28).

A gene overexpressed upon adaptation to ampicillin and* n-*butanol, *wzc*, is part of the colanic acid gene cluster. Colanic acid is an external polysaccharide that is an important component of the cell wall in a number of bacteria, including *Salmonella* and *Klebsiella* spp. (29), and the gene cluster has been implicated in response to ampicillin treatment, resistance to desiccation, and formation of biofilms (16, 29, 30). Other genes in the colanic acid gene cluster, including *wcaA*, *wcaE*, and *gmd*, were also significantly overexpressed in both of the ampicillin-adapted populations (Data Set S1), emphasizing the potential importance of this operon. *amtB*, an ammonia transporter regulated by the sigma factor σ^N^, and *citC*, encoding a citrate lyase synthetase, were both overexpressed upon ampicillin and tetracycline adaptation. Overexpression of *citC* has previously been associated with SOS response induction upon β-lactam exposure, though it is unclear if *citC* plays a role in the SOS response or is merely altered as a consequence of physical proximity on the genome to the DpiBA two-component signal transduction system (31).

Among the genes of unknown function, *yfiL* was found to be differentially underexpressed upon ampicillin and tetracycline adaptation. *yfiL* likely encodes a lipoprotein (32) and has been connected to the general stress response (regulated by the sigma factor σ^s^). Genes in the same operon play a role in motility and persistence in pathogenic *E. coli* and in an analogous operon in *Pseudomonas aeruginosa* (33). Another gene of unknown function, *yjjZ*, was the only gene DE in ampicillin-, tetracycline-, and* n-*butanol-adapted populations (significantly underexpressed in 5 out of 6 of the adapted populations). *yjjZ* has been mentioned as a potential small RNA (34), and so it may perform a regulatory role. Overall, these results indicate that functional classes promoting adaptive resistance include those that conserve energy by reducing motility, implementing protective membrane modifications, and priming cells for mutation. Importantly, the focused set of 11 DE genes are found by examining trends across diverse adaptation conditions and therefore likely represent universal players in adaptive resistance.

### Transcriptome-level signatures based on differential gene expression variability.

**FIG 3:**
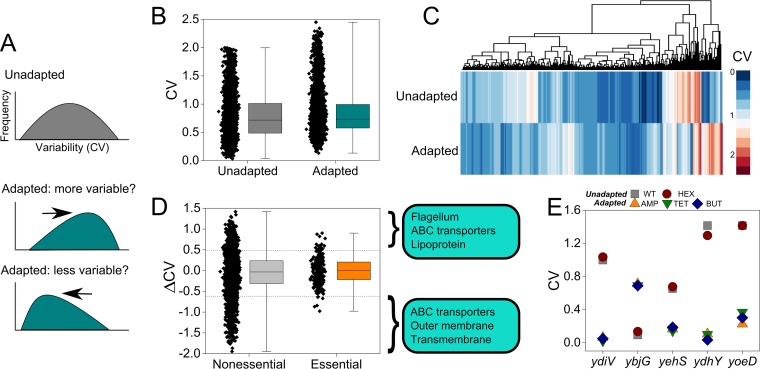
Shifts in gene expression variability are present during bacterial adaptation. (A) Hypothetical distribution in interpopulation gene expression variability. If unadapted samples possess a certain distribution, we predict that shifts in variability will occur in adapted populations. (B) Distribution of variability (CV in FPKM) in gene expression across 4,181 genes in unadapted and adapted samples. For box plots in panels B and D, all data points are shown for each condition. Box plots display the interquartile range and median for the corresponding data. Whiskers on box plots show the minimum and maximum values. (C) Hierarchical clustering by gene expression variability reveals clusters of genes (on vertical axis) with higher and lower variability in unadapted versus adapted bacterial populations. (D) Shifts in gene expression variability in nonessential and essential genes. Shifts are defined as ΔCV = CV_unadapted_ − CV_adapted_. For ΔCV < 0, the gene has higher expression variability in adapted populations. For ΔCV > 0, the gene has lower variability in adapted populations. The three most enriched gene ontologies are displayed for the 10% of genes with highest and lowest ΔCV (10th and 90th percentiles in ΔCV for all genes are marked with horizontal dashed lines). (E) CV across duplicates for five genes with significantly different expression variability in adapted versus unadapted populations. Abbreviations are as in Fig. 1.

Gene expression variability is becoming increasingly acknowledged as a metric by which to evaluate transcriptome data, providing relevant information for human disease (35, 36) and allowing for predictions of a gene’s connectivity in a regulatory network (37, 38). We have previously recognized that measuring expression variability in *E. coli* stress response genes granted a measure for a gene’s involvement in adaptive resistance, with lower-variability genes more likely to impact adaptation (22). Continuing that proposition, we here hypothesized that transcriptome-level shifts in gene expression would be present when comparing unadapted and adapted populations (Fig. 3A). These shifts could point to genes with significant differential variability (DV) and potential relevance for adaptive resistance. To quantify gene expression variability, we compared the variability in normalized transcript abundance (coefficient of variation [CV] = σ/μ in FPKM) across the four unadapted (two hexane and two wild-type populations) to that across all six adapted populations. Overall, there was a significant shift (*P* = 8.6e−11) toward increased gene expression variability across adapted versus unadapted populations (Fig. 3B), which is consistent with the divergence in gene expression (Fig. 1). Hierarchical clustering further underscores the shifts in expression variability at the transcriptome level and underscores sets of genes with similar trends in interpopulation expression variability (Fig. 3C), including genes with higher variability (red) as well as lower variability (blue). To better comprehend the impacts of the variability shifts observable in this transcriptome-level heat map, we calculated the ΔCV for each gene between unadapted and adapted populations and plotted the data in Fig. 3D.

When examining variability according to the essentiality reported in the PEC database (http://www.shigen.nig.ac.jp/ecoli/pec/index.jsp), nonessential genes demonstrated larger shifts in variability than essential genes (Fig. 3D) (*P* = 9.2e−11), suggesting that essential genes are more tightly regulated than nonessential genes. We located enriched gene ontologies in genes (essential and nonessential) with the largest (10th and 90th percentile) shifts in variability for unadapted versus adapted strains (Fig. 3D; Data Set S1) via DAVID v. 6.8 (23). The three most enriched classes for genes exhibiting lower variability upon adaptation (ΔCV > 0) were flagellum (enriched 17-fold, *P* = 1.5e−12), ABC transporters (47-fold, *P* = 4.3e−18), and lipoprotein (37-fold, *P* = 8.1e−14). The three most enriched classes for genes with higher variability upon adaptation (ΔCV < 0) were associated with ABC transport (enriched 49-fold, *P* = 1.5e−16), outer membrane (41-fold, *P* = 3.2e−9), and transmembrane (4.1-fold, *P* = 3.3e−52).

We identified a subset of five genes with significant DV in gene expression by using *t* tests and controlling the false discovery rate with Benjamini and Hochberg’s adjustment (39) (Fig. 3E; Data Set S1). Notably, though the overall trend is toward increased variability, only *ybjG* showed significantly higher variability in adapted populations, whereas the remaining four DV genes (*ydiV*, *yehS*, *ydhY*, and* yoeD*) displayed significantly lower variability across adapted populations. Prior research supports that some of the DV genes influence resistance or stress response; for instance, *ybjG* is a putative bacitracin resistance gene (40), the motility-associated gene *ydiV* (41) is regulated by the membrane stress sigma factor σ^E^, and the general stress sigma factor σ^S^ regulates the predicted oxidoreductase *ydhY* (42, 43). The functions of the conserved protein *yehS* and the pseudogene *yoeD* are unknown (Text S1 contains discussion on the potential roles of these genes).

### Regulatory networks influencing the expression of common DE and DV genes.

An intriguing question is that of the upstream regulation of the DE/DV genes identified here—do these genes point to the relevance of a specific set of regulatory pathways in controlling adaptive resistance? Using information available in EcoCyc (44) and RegulonDB (45), we traced the regulation of the 11 common DE genes and the five DV genes upstream, continuing upstream until no additional regulators were detected. A total of 112 regulatory genes were identified that are known to either directly or indirectly regulate one or more of the target genes (Data Set S1). Overall, we note the influence of many different types of regulators, including 63 transcription factors (e.g., *oxyR*, associated with oxidative stress, and *marA*, mostly commonly associated with antibiotic treatment), 22 genes involved with signal transduction (e.g., AcrAB and EnvZ/OmpR two-component signaling systems), 17 small RNAs (including *arcZ*, *chiX*, and *micA*), and four sigma factors (σ^S^, σ^E^, σ^H^, and σ^N^), all of which present potential targets for future studies attempting to control adaptive resistance at the nongenetic level.

**FIG 4:**
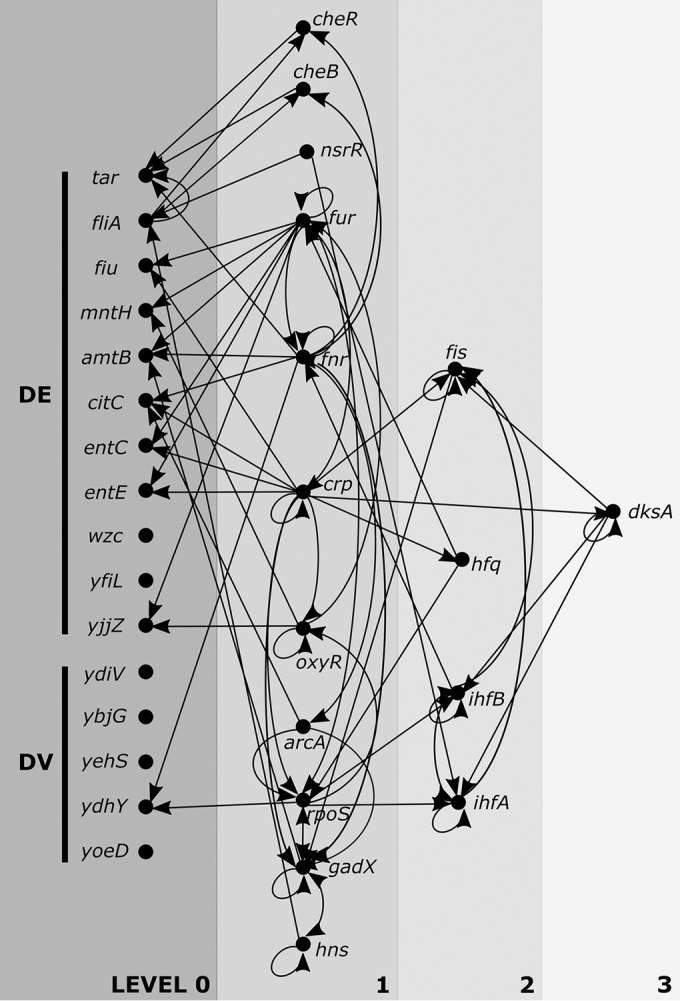
Upstream regulators of target genes. The diagram shows a simplified version of the network regulating target DE and DV genes. Here, only regulators that control two or more of the target genes (in level 0) are shown. Proceeding upstream (moving left to right), only regulators that control two or more of target genes and lower-level genes are included. Arrows indicate the direction of the regulation (i.e., *cheR* regulates *tar*).

To further mine critical regulators within the set of 112 genes, we present a simplified analysis of this network in Fig. 4, where in level 1 we include only the genes known to regulate or be regulated by two or more of the DE/DV genes. Proceeding upstream, additional levels 2 and 3 of regulation include genes that further control or are controlled by two or more of the target genes or their regulators. This simplified analysis identifies a total of 16 regulatory genes, including 11 genes at level 1 (*cheR*, *cheB*, *nsrR*, *fur*, *fnr*, *crp*, *oxyR*, *arcA*, *rpoS*, *gadX*, and *hns*) and 4 genes at level 2 (*fis*, *hfq*, *ihfB*, and *ihfA*), finally peaking with a single gene (*dksA*) at level 3. *dksA* is an RNA polymerase-binding regulator that works with the alarmone ppGpp to control rRNA promoters and others with unstable open promoter complexes, most commonly associated with a starvation response (46, 47). *dksA* has 5 connections in the network—regulating *fis*, *ihfA*, *ihfB*, and itself while being regulated by *crp—*and likely represents an important node of regulation.

The average number of connections among genes in levels 1 to 3 is 6.1 ± 3.2. *nsrR* has the fewest connections, at 2. The most highly connected regulators within the set include the Fur transcriptional dual regulator, the fumarate and nitrate reductase DNA-binding transcriptional dual regulator (FNR), and the cyclic AMP receptor protein (CRP), as well as the sigma factor σ^S^ (encoded by *rpoS*). Fur, which regulates iron homeostasis, has been established to influence the evolution of *de novo* resistance to ciprofloxacin, potentially by increased mutagenesis spurred by iron overload (48). Of the DE/DV genes, Fur regulates *amtB*, *yjjZ*, *fiu*, *mntH*, *entC*, and *entE*, in addition to influencing the expression of the regulators *rpoS* and *fnr* and being influenced by *crp*, *hfq*, and itself for a total of 11 connections. The primary role for FNR is to enable the transition to anaerobic metabolism, though transcriptome analysis has revealed that FNR regulates genes involved with a variety of functions, including chemotaxis, osmotic adaptation, and transport (49). Here, FNR also has 11 connections: regulating *amtB*, *citC*, *tar*, and *ydhY* of the DE/DV genes and *acrA*, *cheB*, *cheR*, and *gadX* of other regulators and being regulated by itself, *acrA*, *fur*, and* ifhB*. Similarly, CRP and σ^S^ each regulate hundreds of genes with a variety of functions. CRP has been discovered to be involved in the stringent response, biofilm formation, and multidrug efflux (50–52) and here has 11 connections. σ^S^ is involved with plasmid transfer and stress-induced mutagenesis (53, 54), in addition to managing gene expression during stationary phase, and has 10 connections.

### Contribution of mutations to gene expression variation.

Genetic variation is expected during adaptive evolution and is likely necessary to ensure heritable resistance. To establish whether the observed gene expression changes can be attributed to mutation, we searched the sequencing data for the presence of variants that could be a factor in DE/DV. None of the five DV or 11 common DE genes contained mutations. Further, none of the alternate sigma factors (σ^S^, σ^E^, σ^N^, σ^F^, σ^H^, and σ^I^) contained mutations in any population. We checked the sequences of 22 transcription factors that directly regulate one or more of the abovementioned genes, including FlhC/D, OxyR, Fnr, Crp, and others. Again, there were no instances in which we could attribute gene expression changes to a change at the genome level (Text S1; Fig. S4). Thus, while mutations in other genes or in intergenic regions may be responsible for expression changes in some populations, it is likely that separate adapted populations have achieved similar gene expression patterns via unique mutations, through the use of nongenetic regulatory pathways, or using a combination of the two. These results indicate that the DE or DV genes would not have been identified through a genome-level mutational analysis.

10.1128/mSphere.00009-17.5 FIG S4  Analysis of genomic-level changes in target genes and their regulators. (A) List of transcription factors that are identified to regulate the indicated differentially expressed or differentially variable genes. We searched the list of variants called to identify whether any of these transcription factors were mutated in any population. (B) Sanger sequencing was performed on the variants called (in RNA sequencing data) in *fiu*, *tyrR*, *wzc*, and *entE* in the indicated sample. In all instances, Sanger sequencing results showed the wild-type sequence. Here, the wild-type codon containing the called variant is shown for each gene. The two sequences below each are extracted from Sanger reads of approximately 400 nt. Download FIG S4, TIF file, 2.1 MB. Copyright © 2017 Erickson et al. 2017 Erickson et al. This content is distributed under the terms of the Creative Commons Attribution 4.0 International license.

10.1128/mSphere.00009-17.6 FIG S5  qPCR validation of CRISPRi. qPCR was performed as described in Materials and Methods. Values of <1 indicate decreased gene expression with respect to the RFP-i control. Horizontal bars indicate the SD from the mean. Download FIG S5, TIF file, 0.04 MB. Copyright © 2017 Erickson et al. 2017 Erickson et al. This content is distributed under the terms of the Creative Commons Attribution 4.0 International license.

10.1128/mSphere.00009-17.7 TABLE S1  Plasmids used in the study. Download TABLE S1, DOCX file, 0.01 MB. Copyright © 2017 Erickson et al. 2017 Erickson et al. This content is distributed under the terms of the Creative Commons Attribution 4.0 International license.

10.1128/mSphere.00009-17.8 TABLE S2  Primers for qPCR, Sanger sequencing, and cloning. Download TABLE S2, DOCX file, 0.01 MB. Copyright © 2017 Erickson et al. 2017 Erickson et al. This content is distributed under the terms of the Creative Commons Attribution 4.0 International license.

### Application of CRISPRi to assess impact of target genes on adaptation.

**FIG 5:**
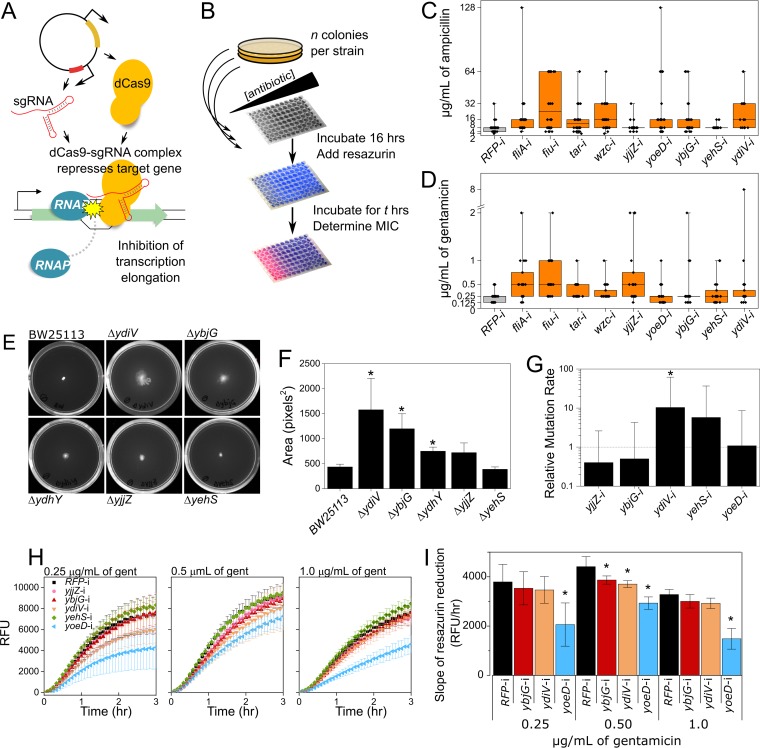
Synthetic perturbation of DE and DV genes. (A) CRISPR interference (CRISPRi) is used to repress gene expression by blocking progression of RNA polymerase (RNAP) at a site specified by the sequence of the sgRNA. The dCas9 protein and the sgRNA are expressed from a medium-copy-number plasmid. (B) MIC was determined for individual colonies from each CRISPRi strain. Colonies were grown for 16 h in a range of antibiotic concentrations, and MIC was determined visually through a resazurin assay. (C and D) The MICs of ampicillin (C) and gentamicin (D) are shown for individual colonies from each CRISPRi strain. Box plots show the interquartile range. The median is marked with a horizontal line. Whiskers demarcate minimum and maximum values. Individual data points are overlaid on the box plots. *n* = 19 to 50 colonies per strain. (E) Representative plates from swarming motility assay, for *E. coli* BW25113 wild-type and five knockout strains after 48 h of growth. (F) Average area from swarming motility assay. Error bars represent the standard deviation across *n* = 3 replicates per strain. (G) Relative mutation rates for CRISPRi strains (rate of strain/rate of RFP-i control). Error bars represent the standard deviation (*n* = 30 parallel cultures for each). (H) Resazurin reduction curves. RFU, relative florescence units. Error bars are the standard deviation (*n* = 4 replicates). gent, gentamicin. (I) Slopes of resazurin reduction curves in panel H. For panels F, G, and I, asterisks indicate a result significantly different from the control (*P* < 0.05).

The DE and DV genes presented here may offer interesting targets for attempts to impede adaptive resistance mechanisms. To validate our approach for identifying key players in adaptive resistance and demonstrate that these genes are involved with adaptation, we applied an engineered type II CRISPR-Cas system to perturb gene expression (Fig. 5A). CRISPR interference (CRISPRi) systems are well suited to mimic natural cellular responses, as they allow for precise manipulation of target gene expression (55, 70). We transformed *E. coli* MG1655 with a plasmid expressing an inducible deactivated type II Cas9 protein (dCas9) and a single guide RNA (sgRNA) targeting one of a randomly selected set of five DE (*fiu*, *fliA*, *tar*, *wzc*, and *yjjZ*) and four DV (*yoeD*, *ybjG*, *yehS*, and *ydiV*) genes. As a control, we included a plasmid expressing an sgRNA targeting a red fluorescent protein (RFP) not present in *E. coli* MG1655. Colonies from each strain (here referred to by the gene that is targeted and “-i” to represent interference) were subjected to a range of antibiotic concentrations, and a visual resazurin assay (56) was used to ascertain the MIC for each colony (Fig. 5B to D). The MIC as well as the degree of heterogeneity introduced in the MIC was used as an indicator for each gene’s involvement in adaptive resistance.

When establishing the MIC of ampicillin (Fig. 5C) or gentamicin (Fig. 5D), perturbation of the target genes generally resulted in increased intrastrain heterogeneity in MIC. The range in MIC increased (relative to the control) in seven out of the nine strains. As the ability to generate diversity is a hallmark of adaptation (5, 14, 57), this finding is a strong indicator that both DE and DV genes influence adaptive resistance. We used one-way analysis of variance (ANOVA) and Bonferroni tests to determine whether significant differences existed in the average MIC. In ampicillin (Fig. 5C), both *yoeD*-i (*P* = 0.006) and *fiu*-i (*P* = 8.6e−7) had significantly higher average MICs than the control. The MIC of *fiu*-i was also significantly higher than that of *tar*-i, *yjjZ*-i, *ybjG*-i, and *yehS*-i (*P* < 0.03 for all), while *yoeD*-i had a higher MIC than *yjjZ*-i (*P* = 0.02).

While none of the strains exposed to gentamicin had a significantly higher average MIC, the trend of increased heterogeneity was maintained (Fig. 5D). Furthermore, increased heterogeneity in MIC is associated with higher frequency of clinically relevant resistances. For instance, the Clinical and Laboratory Standards Institute (CLSI) sets an ampicillin resistance breakpoint at ≥32 μg/ml of ampicillin (58). By this standard, one of the 50 (2%) *RFP*-i colonies was resistant to ampicillin. In contrast, resistance was achieved in >20% of *fliA*-i, *fiu*-i, *wzc*-i, *yoeD*-i, and *ydiV*-i colonies. The *fliA*-i and *yoeD*-i strains in particular demonstrated the greatest range of ampicillin MICs, with colonies in each having MICs as low as 4 or as high as 128 μg/ml of ampicillin (Fig. 5C). The CLSI breakpoint for gentamicin resistance is ≥16 μg/ml. Although no colonies were gentamicin resistant (Fig. 5D), the general trend of increased frequency of higher MIC was maintained. Fifteen percent of control RFP-i colonies had a MIC of ≥0.5 μg/ml of gentamicin, while all of the CRISPRi strains had a higher proportion of colonies with a MIC at or above 0.5 μg/ml, including 60% of *fliA*-i, 70% of *fiu*-i, and 55% of *yjjZ*-i colonies. As there were significant differences between CRISPRi strains, with *yjjZ*-i and *yehS*-i especially having ampicillin MIC distributions similar to the control, we can deduce that these results are attributable to the specific perturbation of the target gene and not merely to any disruption of normal cellular function. These data demonstrate that subtle expression changes in certain genes can impact the likelihood of survival in the presence of high levels of stress, providing a more favorable environment in which to develop heritable resistances.

### Certain target genes impact swarming motility.

As our gene ontology enrichment analysis identified many motility-associated changes in DE or DV genes, we sought to determine whether DE/DV genes of unknown function also influence adaptation through a motility-associated mechanism. We obtained gene-knockout strains for the DV genes *ydiV*, *ybjG*, *ydhY*, and *yehS* and the DE gene *yjjZ* and then compared the motility to that of the wild-type strain *E. coli* BW25113. Figure 5E shows a representative image of each strain after 48 h of growth on semisolid agar plates (M9 minimal medium with 0.3% agar). We find significant increases in motility in the Δ*ydiV*, Δ*ybjG*, and Δ*ydhY* strains (Fig. 5F). Overexpression of the anti-FlhDC factor *ydiV* has been previously shown to decrease motility (41), in agreement with our findings. However, neither *ybjG* nor *ydhY* has been previously shown to influence motility. While it is not straightforward to rationalize how changes in variability of these genes might be reflected in a phenotype, our results suggest that shifts in variability of *ydiV*, *ybjG*, and *ydhY* could lead to phenotypic heterogeneity in motility, in turn promoting survival in the presence of stress.

### Mutation rates in CRISPR interference strains.

Increased mutation rates could be a mechanism for higher and more heterogeneous MICs. We performed standard fluctuation tests to assess whether CRISPRi influences mutation rates. For four out of the five CRISPRi strains evaluated, we found that the mutation rates were not significantly different between CRISPRi strains and the control (Fig. 5G). Therefore, we can conclude that the CRISPRi system does not inherently increase mutation rates independently of the gene being targeted and that unintentional increases in mutation rate are not the likely explanation for the phenotypic heterogeneity present in CRISPRi strains like *yoeD*-i, *ybjG*-i, and *yjjZ*-i. Interestingly, we did observe that the *ydiV*-i strain has a mutation rate significantly higher than the RFP-i control (10-fold higher). As mentioned above, *ydiV* does have a known function as an anti-FlhDC factor. Our results suggest that it may impact mutation rates as well, though further investigation is needed to elucidate the precise mechanism by which *ydiV* contributes to adaptive resistance.

### Metabolic rates in CRISPR interference strains.

Our gene ontology enrichment analysis also revealed a differential expression in a multitude of genes associated with metabolism. Thus, the DV and DE genes of unknown function could potentially impact adaptation by promoting changes in metabolism. Resazurin dye is reduced to the fluorescent resorufin through an irreversible reaction catalyzed by dehydrogenases and dependent upon NADH availability (59). Therefore, by adding resazurin to cultures and monitoring the changes in fluorescence over time, high-level insight into metabolic rates within populations can be garnered. We used a resazurin reduction assay to measure metabolic rates in CRISPRi strains subjected to a range of gentamicin concentrations (Fig. 5H). We find that *yoeD*-i has a consistently decreased metabolic rate as measured by the slope of the resazurin reduction curve for a range of gentamicin concentrations, including 0.25 µg/ml (*P* = 0.02), 0.5 µg/ml (*P* = 0.0008), and 1 µg/ml (*P* = 0.0003) of gentamicin (Fig. 5I). The average metabolic rate of *yoeD*-i strains was reduced by 46% (compared to the control) in 0.25 µg/ml of gentamicin, by 34% in 0.5 µg/ml of gentamicin, and by 55% in 1 µg/ml of gentamicin. None of the other strains had metabolic rates reduced to such an extent or reduced in more than one concentration, though *ybjG*-i and *ydiV*-i had slightly reduced rates in 1 µg/ml of gentamicin (12% reduction, *P* = 0.05, and 16% reduction, *P* = 0.02, respectively).

### Conclusions.

Here, we compare transcriptome patterns in heterogeneous adapted and unadapted bacterial populations in order to locate key genes and pathways contributing to adaptive resistance. While others have used mutant library selection approaches to detect genes which convey specific tolerances or resistances (60, 61), only transcriptome profiling allows for the detection of subtle and simultaneous changes across multiple genes. Ascertaining general signatures of adaptation is not trivial, due to the immense potential for heterogeneity in gene expression during adaptation (4, 5, 8). In this study, by intentionally generating diversity at the phenotypic as well as gene expression level via medium-term adaptation to diverse toxins, we identified a subset of 16 genes with significantly different expression characteristics across multiple adaptation conditions. Many of the target genes are supported by previous reports, though several are of unknown function, particularly those genes identified via differential variability analysis. The DE and DV genes suggest the importance of changes in motility, metabolism, membrane structure, and transport during adaptation to diverse conditions. This study also emphasizes global regulators potentially linked to adaptation, which were not themselves DE or DV but were recognized by examining the upstream regulation of the DE/DV genes. Locating key regulators may not always be possible through DE/DV analysis alone; for instance, FNR has similar expression levels under anaerobic and aerobic conditions and is activated only when oxygen induces a conformational shift (62). Therefore, an analysis of the known regulators of genes identified through a top-down approach is necessary to garner a more complete understanding of the regulation of adaptive resistance.

Importantly, this work substantiates the idea that bacterial adaptation is enabled not only by changes in gene expression levels but also by shifts in gene expression variability. Gene expression variability analysis is emerging as a powerful method, particularly in eukaryotic systems, but is not often incorporated into bacterial transcriptome analysis. For instance, in human stem cells and in yeast, genes trending toward lower variability were found to be more likely to be essential or highly connected (i.e., to play a regulatory role) (37, 38, 63). Our whole-transcriptome variability analysis is in line with those performed in eukaryotic systems—essential genes experienced lower magnitudes of variability shifts upon adaptation than did nonessential genes. In this study, the observation that variability shifts occur during adaptation is also consistent with previous studies in yeast, which have demonstrated that expression “noise” is a selectable trait (64). We find an overall shift toward increased variability in adapted versus unadapted populations. This could be attributed to the fact that different gene expression states are being selected for across divergent populations but could also be due to intrinsic regulation of an adaptive response, considering that stress response genes have been found to tend toward higher variability in mice and yeast (65, 66). In this study, five genes with significantly different gene expression variabilities were located, four out of five of which had decreased variability upon adaptation. We have previously suggested that shifts toward lower variability may impart evidence of involvement with adaptation (22), and the transcriptome-level validation here implies that gene expression variability is tunable in bacteria as well as eukaryotic systems. We postulate that genes involved with the transient adaptive resistance process likely have differential variability between unstressed and stressed conditions. In our data, we observe that the majority of the DV genes demonstrate a shift toward tight regulation and lower variability only upon the addition of stress. Theoretical models of the *mar* regulon in *E. coli* support this theory; a high-noise state was found to be lower cost, while the addition of salicylate produced a low-noise, higher-cost state (67). Further studies, especially in bacterial systems, will enable the field to decipher the complex regulation of expression variability in regard to evolutionary responses.

When expression of DE and DV genes was perturbed with CRISPRi, we found increased prevalence of higher MICs as well as larger heterogeneity in MICs in both DE and DV gene targets. Indeed, many of the perturbed strains had MIC profiles more closely resembling those associated with endpoint adapted populations (22). We briefly investigated the mechanisms by which the DV genes of unknown function could be contributing to adaptive resistance. While most CRISPRi strains had mutation rates similar to that of the control strain, perturbation of *ydiV* increased mutation rates approximately 10-fold, a function that has not been previously attributed to this motility-associated gene. Two other DV genes, *ybjG* and *ydhY*, also appear to impact motility, providing further evidence to support that regulation of motility is important to a multitude of stress response pathways (68). Finally, we observed that perturbation of *yoeD* influences metabolic rates in a range of gentamicin concentrations, suggesting a metabolic or global regulatory role for this gene of unknown function.

Our results together support the existence of a nongenetic basis for adaptive resistance; subtle gene expression changes are sufficient to drive increased resistance in bacterial populations. The DE/DV genes presented in this study, as well their regulators, deliver a snapshot of the complex response controlling adaptive resistance. Continued inquiry using approaches similar to those presented here, and expanding to investigate additional stress conditions and bacterial species, will only further our understanding and ability to impede the upstream, nongenetic responses that enable the eventual emergence of novel antibiotic resistances.

## MATERIALS AND METHODS

### Strains and culture conditions for adaptation experiments.

*E. coli* K-12 strain MG1655 (ATCC 700926) was used in adaptation experiments. Unless otherwise mentioned, all strains were propagated in M9 minimal medium (5× M9 minimal medium salts solution from MP Biomedicals, 2.0 mM MgSO_4_, and 0.1 mM CaCl_2_ in sterile water) with 0.4% glucose. Strains were adapted to ampicillin, tetracycline, or* n-*butanol or grown in* n-*hexane as described previously (22). Briefly, cultures were propagated via serial dilution (1:5 to 1:100 dilutions, depending on OD at 600 nm [OD_600_]) in increasing concentrations of toxin until either the MIC had increased to four times the initial MIC or until no resistance gains were observed for seven consecutive 24-h growth periods. OD_600_ was measured on a NanoDrop 2000 spectrophotometer (Thermo Scientific), using 2 μl of culture. Cultures were considered resistant to a certain concentration of toxin if the OD_600_ was ≥0.5 after 24 h of growth at 37°C. Bacterial cultures (500 μl) with an OD_600_ of >0.54 and <0.84 were mixed with 1 ml RNAprotect bacterial reagent (Qiagen), flash-frozen in dry ice and ethanol, and stored at −80°C until RNA extraction. Glycerol stocks were prepared by spinning down 0.5 ml of culture at 4,000 rpm for 5 min, pouring off the supernatant, and then resuspending the remnant in LB with 50% glycerol. Stocks were stored at −80°C. Populations sequenced correspond to wild-type populations 1 and 2, ampicillin populations 2 and 3, tetracycline populations 1 and 2, butanol populations 1 and 2, and* n-*hexane populations 1 and 3 from the related publication (22).

### RNA sequencing library preparation.

Total RNA for sequencing was extracted using phenol-chloroform extraction with a TRIzol Max bacterial RNA isolation kit (Ambion). RNA was treated with the Turbo DNA-free kit (Ambion) to remove DNA. RNA concentration and *A*_260_/*A*_280_ ratios (>1.8) were obtained with a NanoDrop 2000 spectrophotometer (Thermo Scientific). rRNA treatment and library preparation were carried out at the Genomics and Microarray Core Facility (Anschutz Medical Campus, University of Colorado Denver). Ten sequencing libraries were prepared using 80 to 600 ng of total RNA per sample and nonstranded Nugen kits. All samples were sequenced in one lane of an Illumina HiSeq 2000 with 1 × 100-bp reads, generating an average of 28.6 ± 2.2 million reads per library.

### Sequencing data analysis.

The *E. coli* K-12 MG1655 reference FASTA and gene annotation files were obtained from Ensembl, in the bacteria_22 collection (files Escherichia_coli_k_12_substr_ mg1655.gca_000005845.2.22.dna.chromosome.Chromosome.fa and Escherichia_coli_k_12_substr_mg1655.gca_000005845.2.22.chromosome.Chromosome.gff3). The TopHat/Cufflinks workflow (69) was used to identify differentially expressed transcripts and to calculate FPKM for differential variability analysis.

Further details on the sequencing data analysis, growth characterization, fluctuation tests, motility assays, statistical analysis, CRISPRi, and other experiments described are provided in Text S1 in the supplemental material.

### Accession number(s).

Data have been deposited in NCBI’s Sequence Read Archive (accession no. SRP069322).
