# Supplementary material for: Transcriptome-Level Signatures in Gene Expression and Gene Expression Variability during Bacterial Adaptive Evolution
Source: mSphere. 2017 Feb 15;2(1):e00009-17. doi: 10.1128/mSphere.00009-17 (PMC5311112; doi:10.1128/mSphere.00009-17)
Supplement: TEXT S1 [file sph001162232s1.pdf]

Supplemental Materials for

Transcriptome-level signatures in gene expression and gene expression variability during bacterial adaptive evolution

Keesha E. Erickson, Peter B. Otoupal, Anushree Chatterjee*

*Corresponding author. Email: chatterjee@colorado.edu

This file includes:

Supplemental Materials and Methods

Supplemental Text

Supplemental References

Supplemental Materials and Methods

Bacterial growth curves. Bulk cultures were inoculated from glycerol stocks of either *E. coli* MG1655 or adapted populations into microplate wells containing 100 µL of fresh M9 media with or without corresponding selection pressure. Ampicillin-adapted populations were re-grown in media with 100 µg/mL ampicillin. Tetracycline-adapted populations were re-grown in media with 2 µg/mL tetracycline. Butanol adapted populations were re-grown in media with 0.5% v/v n-butanol. Growth was measured using a Tecan GENios plate reader (Tecan Group Ltd.) with Magellan™ software version 7.2. Absorbance was read at 562 nm and 37^0C every 20 minutes, with shaking between measurements. To compare optical densities obtained from the microplate reader to those obtained from the Nanodrop, samples of varying optical density were measured on each machine, and used to obtain a conversion factor.

Sequencing data analysis. TopHat version 2.0.6 was applied to map reads to the Ensembl reference files, then the Cufflinks software version 2.1.1 was applied to assemble transcripts (via Cufflinks), combine transcript assembly files (via Cuffmerge), and calculate differential expression (via Cuffdiff). To account for differences in rRNA treatment during sample preparation, the mask option was used to remove the ribosomal RNA transcripts prior to calculating differential expression. Bias correction and multi-read mapping correction options were also applied. As an additional quality check, we compared expression levels obtained from Cufflinks and DESeq (3) to levels obtained from qPCR (Fig. S2). To investigate differential expression in each of the eight individual sample populations, calculations were performed with partial replicates: two wild type populations considered to be biological replicates were used together to separately analyze each of the additional eight populations. Libraries were normalized by fragments per kilobase of transcript per million mapped reads (FPKM), variance

was estimated with a pooled dispersion method, and genes were considered to be significantly DE if *P*<0.05 and *q*<0.30. To estimate differential gene expression variability (DV), the coefficient of variation (CV) for adapted and unadapted samples was calculated from the FPKM for each gene. Genes for which FPKM values were flagged by Cufflinks to have low or high data were removed prior to analysis, as were genes with a mean FPKM=0 across any two set of duplicates, and four genes that had FPKM=0 in at least one replicate in all conditions. A two-tailed type two Student's t-test was used to compare the CV across three adapted and two unadapted growth conditions. The false discovery rate was controlled with Benjamini and Hochberg's algorithm (4). Genes were considered significantly DV if *P*<0.05 and *q*<0.30.

Furthermore, we examined the differentially expressed genes and transcript abundances for a correlation with distance from the origin, which would indicate that minor differences in optical density at time of sampling are resulting in different growth phases that impact the results of gene expression analysis. We compared the normalized expression values from all differentially expressed genes to their chromosomal position and their absolute distance from the origin. The Pearson correlation coefficient for chromosomal position versus expression value was -0.010 with a *P*-value of 0.71. The correlation coefficient for distance from origin versus expression values was -0.023 with a *P*-value of 0.41. We also examined all genes in each library using FPKM vs chromosomal location or distance from origin, and found no significant correlation between position and transcript abundance for any of the libraries. Pearson correlation coefficients ranged from -0.015 to 0.029 with *P*-values from 0.066 to 0.95. Thus, all correlation coefficients strongly indicate that differential expression observed in this work cannot be explained by chromosomal position or proximity to the origin.

Bowtie 2 version 2.0.2 (5) was used to index the reference genome and generate alignment (sam) files using end-to-end alignment mode and default scoring from each FASTQ file. Alignment sam files were converted to sorted bam files using SAMtools version 0.1.18 (6). Indels and single nucleotide polymorphisms (SNPs) were called using the Genome Analysis Toolkit version 2.4-9(7). Variants were called from sorted bam files from which PCR duplicates had been removed with SAMtools. SNP calls were filtered according to quality by depth (QD<2.0), mapping quality (MQ<40.0, MappingQualityRankSum< -12.5), strand bias (FS>60), and position of alternate allele in the read (ReadPosRankSum< -2.0). Indel calls were filtered according to quality by depth (QD<2.0), strand bias (FS>60), and position of alternate allele in the read (ReadPosRankSum< -2.0). The ReadPosRankSum requirement was made more stringent according to observations that many of the false positives called were located near the end of reads. A custom Python script was used to add annotations (type of mutation, gene affected, synonymous or non-synonymous, amino acid change) in comparison to the Ensembl reference and gene annotation files. A custom MATLAB script was used to remove variants that exactly matched any variant in wild type samples, and find overlaps between populations. The Integrative Genomics Viewer(8) (IGV) was used to visualize all variants that passed the filter in DE genes, DV genes, and transcription factors regulating DE/DV genes. In many cases, false variants passed the filtering stage (e.g. variant in a minority of reads, and only located at the end of reads). Variants that appeared to be true calls or variant calls that were ambiguous were verified with Sanger sequencing, as follows: Glycerol stocks were streaked on LB agar and grown for 16 hours at 37°C. Two colonies from each population were selected for sequencing (Quintarabio). Colony PCR was performed with Phusion DNA polymerase to amplify an approximately 400 bp region of genomic DNA flanking the purported polymorphism. Amplification was verified with gel electrophoresis.

Bands were extracted with a Zymoclean Gel DNA recovery kit (Zymo). DNA concentration was verified with a Nanodrop 2000. Templates were diluted to 5 ng/µL in nuclease-free water with 5 µM of primer. Wild type and adapted sequences were aligned with UGENE v1.17.0 (http://ugene.net/).

Gene ontology classification. For analysis of enriched gene ontology terms (in Fig. 1D, Fig. 3D, and File S1), we applied DAVID's functional annotation clustering tool (version 6.8) with the highest classification stringency (9). In the main text, we report the fold enrichment and the *P*-value from DAVID's EASE score, a more conservative Fisher's exact *P*-value. For Fig. 1D and 3D, we include the three most enriched functional classifications while in File S1, all unique enriched functional classifications are reported. For DE genes in File S1, we entered the 321 genes identified as significantly differentially under-expressed and the 434 genes significantly over-expressed in at least one of the ampicillin, tetracycline, or n-butanol adapted populations. For variability shifts in Fig. 3D and File S1, we entered 0-10th percentile of ΔCV, including 418 genes that became more variable, and the 90-100th percentile, which included 418 genes that became less variable.

Gene ontology information in Fig. 2 was derived from the Ecocyc and EcoliWiki annotation project file, validation date 10/31/2014 (10). We manually simplified the extensive gene ontology list to 27 categories, and allowed each gene to participate in only one category.

CRISRRi plasmid assembly. A list of plasmids used in this study is provided in Table S1. The CRISPRi control plasmid targeting a DNA sequence not present in bacteria, pRFP-i, was first constructed by cloning the sgRNA portion from pgRNA-bacteria (Addgene plasmid 44251) into the pdCas9 vector (Addgene plasmid 44249) (11). Both plasmids were digested with the restriction

enzymes XhoI and BsrBI (New England Biolabs). The 712 bp digestion product from pgRNA-bacteria was gel-purified using Zymoclean™ Gel DNA Recovery Kit (Zymo Research Corporation). The 6497 bp digestion product from pdCas9 was also gel-purified in the same fashion. These products were ligated together using T4 DNA ligase (New England Biolabs) overnight. Ligations were transformed via electroporation into electrocompetent NEB 10-β cells. Plasmid minipreps were performed using the Zyppy™ Plasmid Miniprep Kit (Zymo Research Corporation). The recovered plasmid was then transformed into chemically competent *E. coli* MG1655, and a miniprep was again performed and sequenced for validation of correct assembly product (GENEWIZ). A digestion confirmation was also performed by digesting the plasmid with EcoRI (New England Biolabs), in which three distinct bands were identified to confirm the product's assembly. The ultimate plasmid product includes aTc-inducible expression of dCas9, a chloramphenicol resistance marker, a constitutively-expressed sgRNA, and the strong rrnB terminator between the ORFs of dCas9 and sgRNA.

All CRISPRi plasmids perturbing target gene expression were derived from pRFP-i. Single guide RNA (sgRNA) targeting sequences were 20 nt long, and located immediately downstream of an NGG protospacer adjacent motif (PAM) near the 5' end of the target open reading frame. This was accomplished by amplifying new sgRNA targets via PCR using Phusion® High-Fidelity DNA Polymerase (New England Biolabs). Primers were designed and ordered from Invitrogen™ Custom DNA Oligos (Thermo Scientific) and are listed in Table S2. A common reverse primer was used, with unique forward primers to replace the 20 nt RFP-complementary sequence with information dictating the new sgRNA sequence. The resulting 124 nt fragments and pgRNA-bacteria plasmid backbone were digested with ApaI and SpeI, then ligated together with T4 DNA ligase. The recovered pgRNA-bacteria plasmids (now with new sgRNA targets) and the pRFP-i

plasmid were digested with XhoI and AvrII (New England Biolabs). The 498 bp new sgRNA inserts and the 6720 bp pRFP-i backbone were gel-extracted and ligated together as previously outlined. Overnight ligations were electroporated into NEB 10-β electrocompetent cells. The plasmid recovery, confirmation, and transfer to *E. coli* MG1655 were analogous to the process outlined above.

For verification of repression, three colonies from each strain were picked from LB agar+Cm plates and inoculated into 1 mL minimal media with Cm for 16 hours at 37^{0}C. Cultures were diluted 1:20 into new media with 10-40 ng/mL anhydrous tetracycline (aTc) and grown for 6 hours, then samples were harvested and stored in two volumes of RNAprotect Bacterial Reagent (Qiagen). RNA was extracted with a GeneJET RNA Purification Kit (Thermo Scientific). DNA contamination was removed with a TURBO DNA-free kit (Ambion). Reverse transcription was performed with a Dynamo cDNA synthesis kit (Life Technologies) and random hexamers. qPCR reactions were prepared with Maxima SYBR Green qPCR Master Mix. Each reaction contained 1 ng of cDNA template, 0.3 μM of each gene specific primer, 12.5 μL of master mix with SYBR green dye and Maxima Hot Start *Taq* Polymerase, 10 nM ROX dye, and water to 25 μL. The cycling protocol was a 10 min initial denaturation at 95^{0}C, then 40 cycles of denaturation (95^{0}C for 15 s), annealing (55^{0}C for 30 s), and extension (72^{0}C for 30 s). qPCR was run on a QuantStudio 6 Real-Time PCR machine in the CU Core Sequencing Facility. Melt curves and no template controls were run for each primer pair to verify specificity. Standard curves were run with cDNA dilutions to check primer efficiency. No RT controls were run to confirm negligible DNA contamination. Two technical replicates were run for each of three biological replicates. Normalized gene expression was calculated according to the $2^{-\Delta\Delta Cq}$ method(12), using *rrsA* and

gyrA as reference genes, and *E. coli* carrying a CRISPRi plasmid targeting RFP expression as a control strain (Fig. S5).

Minimum inhibitory concentration (MIC) for CRISPRi strains. *E. coli* MG1655 colonies harboring CRISPRi plasmids were picked from LB agar+chloramphenicol plates and suspended in 200 μL of M9 minimal media. 25 μL of each colony suspension was added to 25 μL of media to result in 50 μL cultures with 40 ng/mL of aTc, 25 μg/mL of chloramphenicol, and a range of antibiotic concentrations (0.125-4 μg/mL of gentamicin and 2-64 μg/mL of ampicillin). Cultures were propagated at 37°C with shaking. After 16 hours, resazurin dye was added to each well and the plates were incubated at 37°C. MIC was called for each colony as the lowest concentration of antibiotic for which there was no visible color change after 3 hours (ampicillin assay) or 24 hours (gentamicin assay). For colonies that grew at all concentrations, MIC was called as 2x the highest concentration tested. For colonies that grew at no concentrations, MIC was called as the lowest concentration tested. The number of colonies analyzed for each strain ranged between 19 and 50, with the average at 30 colonies for ampicillin tests and 20 colonies for gentamicin tests.

Fluctuation tests. Mutation rates were estimated with fluctuation tests using the method of Luria and Delbruck (13). Single colonies of the control and CRISPRi strains from LB agar plates were used to inoculate 1 mL cultures in LB, which were grown for 16 hours at 37°C. Cultures were diluted to the same OD_{600} according to measurements on a Nanodrop 2000. Normalized cultures were each diluted 1:10,000 into thirty parallel 100 μL cultures of M9 minimal media with 40 ng/mL of aTc and 25 μg/mL of chloramphenicol. Cultures were grown for 24 hours at 37°C. To determine viable cell counts, 2 μL of each of the 30 cultures per strain were pooled and dilutions were plated on LB agar for CFU analysis. The remaining 98 μL of each culture were plated on LB agar with 100 μg/mL of rifampicin. Colonies on rifampicin plates were counted after 48 hours.

Mutation rates and 95% confidence intervals were determined via the Ma-Sandri-Sarkar maximum-likelihood method (14), implemented by the FALCOR web tool (15). Significance was assessed with Student's t-tests using the mutation rates and confidence intervals calculated by FALCOR.

Swarming motility assay. We performed swarming motility assays on semi-solid plates (M9 minimal media with 0.4% glucose and 0.3% agar). We opted to use Keio collection strains (16) and *E. coli* BW25113 obtained from the Coli Genetic Stock Center (http://cgsc.biology.yale.edu/index.php), since these strains would not require aTc induction for 48 continuous hours to ensure a gene perturbation (as the CRISPRi strains would). We picked 5 colonies for each strain from LB agar plates and resuspended in 20 µL of sterile water. Plates were poured and dried for 30 minutes, then 1 µL of each colony suspension was stabbed into the center of each small plate. Three replicates were plated for each strain. Plates were incubated at 37°C for 48 hours. We photographed the plates using a Gel Doc EZ (Bio-Rad, Hercules, CA), and measured the area of the colonies using a custom pipeline in CellProfiler (17). Images were manually cropped, then colonies were detected using the IdentifyPrimaryObjects analysis module, with a global thresholding strategy and robust background thresholding method. Colony area was measured with the MeasureObjectSizeShape analysis module.

Resazurin metabolic rate assay. Overnight cultures in LB were diluted 1:100 into microplate wells with 40 µL LB, 40 ng/mL of aTc, 25 µg/mL of chloramphenicol, and a range of concentrations of gentamicin (up to 2 µg/mL of gentamicin). Four biological replicates were included for each strain. The plate was incubated for 20 hours at 37°C with 225 rpm shaking, then 4 µL of 10x resazurin was added to each well. Changes in florescence were monitored in a Tecan Genios (excitation 485 nm, emission 610 nm) in five minute intervals. The slope of the curve was

determined using a custom MATLAB script. The most linear slope for each replicate was determined using a sliding window of 5 timepoints and an R^2 value (minimum R^2 value for any replicate was 0.98). Slopes were averaged across the replicates, and two-tailed t-tests were used to compare the slope of the RFP-i control to that of each CRISPRi strain.

Additional software tools and statistical analysis. The 'pca' function from MATLAB version R2014b (The Mathworks, Inc., Natick, MA) was used for principal component analysis (PCA). Correlation coefficients and PCA were performed on FPKM generated through the Cufflinks pipeline. Genes flagged as having low or high data in any one sample were removed from all samples prior to analysis.

The 'clustergram' function from MATLAB's Bioinformatics toolbox was used to perform hierarchical clustering. Dendrograms were built with a Euclidean distance metric, optimal leaf ordering, and average linkage function.

Box plots were generated and other statistical analysis were performed with OriginPro 9.1 software (OriginLab Corporation, Northampton, MA). To compare variability in unadapted and adapted populations, the CV for each gene across wild type and n-hexane populations was compared to the CV for each gene across ampicillin, tetracycline, and n-butanol adapted populations. Significance was calculated with a two-tailed, type two t-test. To compare variability shifts in essential versus non-essential genes, the $|\Delta CV|$ was calculated as $|CV_{unadapted} - CV_{adapted}|$. A two-tailed, type two t-test was used to compare the $|\Delta CV|$ for 288 essential genes to that for 3828 non-essential genes. Genes not in the PEC database (http://www.shigen.nig.ac.jp/ecoli/pec/index.jsp) were excluded from this analysis.

The coefficient of variation for unadapted and adapted samples is the mean of FPKM across all unadapted or adapted populations respectively divided by the standard deviations across the same samples.

To examine the function of unknown genes, nucleotide BLAST was executed through NCBI's web interface at http://blast.ncbi.nlm.nih.gov/. We compared sequences from *E. coli* K12 MG1655 genes to the NCBI Chromosome database with the megablast algorithm (optimized for highly similar sequences).

Supplemental Results & Discussion

Similarities & differences in gene expression levels for tetracycline adapted populations. Using the log2(fold change) values depicted in Fig. 1C, there were 172 genes with ≥2-fold higher expression, and 218 genes with ≥2-fold lower expression in tetracycline population 1 but not 2. Within this set, histidine biosynthesis genes (e.g. *hisA*, *hisB*, *hisD*, *hisH*, *hisI*), membrane proteins (e.g. *yjjG*, *yohK*), and transporters (e.g. *mdtD*, *mdtG*, *dcuC*, *yaaJ*) were over-expressed, while regulators (e.g. *soxS*, *envR*, *higA*) and other transporters and membrane proteins (e.g. *glcA*, *yqeG*, *mdtJ*) were under-expressed.

There were 235 genes with ≥2-fold higher expression in and 251 genes with ≥2-fold lower expression in tetracycline population 2 but not 1. *iraD*, which controls σ^S levels, was one of the over-expressed genes in population 2. Others included various transporters (*dppB*, *trkG*, *sdaC*) and redox associated genes (*cyoABCDE*, *nuoA*).

In both tetracycline populations, 324 genes were ≥2-fold higher and 684 genes were ≥2-fold lower. Genes over-expressed in tetracycline populations 1 and 2 included the multiple antibiotic

resistance regulators *marA* and *marR* as well as several uncharacterized transcriptional regulators (*yagI*, *ydbO*, *yfiR*), pilus-associated genes (*fimA*, *fimG*, *fimH*, *fimI*, *fimF*), electron transport genes (*nuoHIJKLMN*, *rsxABCDE*), and many transport/membrane associated genes. Notable under-expressed genes shared by both populations included motility genes (e.g. *flgKLMN*, *fliCDEF*, *flhAB*, *motAB*) and transcriptional regulators (e.g. *bglJ*, *gadX*, *exuR*, *csiR*).

Similarities and differences in gene expression levels for n-butanol adapted populations. In n-butanol adapted population 1, there were 156 genes over-expressed and 250 genes under-expressed (≥2-fold either way) that were not so in population 2. Over-expressed genes included iron transport genes (*fecCD*), other transporters (*yehY*, *mhpT*), and thiazole biosynthesis genes (*thiH*, *thiG*). A multitude of transporters and membrane proteins were under-expressed (*mdtJ*, *ydhP*, *rcnA*, etc), as were transcriptional regulators (e.g. *ybdO*, *ydcI*, *ytfH*).

In n-butanol adapted populations 2, there were 282 over-expressed genes and 402 under-expressed that were unique from those affected in population 1. Over-expressed genes included a plethora of transporters (e.g. *proP*, *mdtI*, *mdtM*), many uncharacterized lipoproteins (e.g. *yifL*, *yjbH*, yeaY), regulators (e.g. *soxS*, *acrR*, *ybaO*), and colonic acid biosynthesis genes (e.g. *wcaA*, *wcaE*, *wcaJ*). Under-expressed genes included motility genes (e.g. *tar*, *cheA*, *motA*), transcriptional regulators (e.g. *dksA*, *eutR*, *mlrA*), iron transport and metabolism genes (e.g. *fepABC*, *fhuCD*, *fecA*).

The set of 167 genes that were over-expressed in both n-butanol populations included electron transport genes (e.g. *nuoKL*, *ysaA*, *rnfC*), nickel transport genes (*nikABCDE*), histidine biosynthesis genes (e.g. *hisAB*, *hisHI*) and other transporters (e.g. *dppC*, *thiQ*, *proQ*, *oppF*). The 529 genes with decreased expression in both populations were commonly associated with motility

(e.g. *flhAB*, *fliS*, *fliCD*), transcriptional regulation (e.g. *melR*, *feaR*, *exuR*, *bglJ*), and transport (e.g. *gspA*, *ydcT*, *sdaC*, *mdtO*).

Mutation analysis. We called mutations in the RNA-sequencing data using a pipeline described in the Materials & Methods. We used IGV(8) to determine whether a mutation was called in any of the eleven commonly differentially expressed genes (*tar*, *fiu*, *mntH*, *wzc*, *citC*, *entC*, *entE*, *fliA*, *amtB*, *yfiL*, and *yjjZ*), the five differentially variable genes (*ydiV*, *ybjG*, *yehS*, *ydhY*, and *yoeD*), as well as the alternate sigma factors (σ^S, σ^E, σ^N, σ^F, σ^H, and σ^I) and twenty-two transcription factors known to regulate the differentially expressed or variable genes (Fig. S4A). Out of all genes examined, mutations were called in five cases: in *fiu* and *tyrR* in ampicillin population 1, in *wzc* in ampicillin population 2, in *entE* in butanol population 2, and in *tyrR* in tetracycline population 2 (Fig. S4B). We picked 2 colonies from the respective populations and used Sanger sequencing to examine areas of approximately 400 nt, including the called mutation (primers in Table S2). In all instances, the called variant does not appear in Sanger sequencing, suggesting that the called variants emerged during the library preparation or sequencing. Thus, there are no mutations in the common differentially expressed genes or the differentially variable genes, nor any mutations in known regulators of said genes. While there could be mutations in other unknown regulatory regions that promote changes in gene expression, it is clear that attributing gene expression changes to genomic changes is not straightforward. However, analyzing the significance of the gene expression changes can provide insight in the nature of the adaptive transcriptome.

Role of target genes with unknown function. We applied NCBI's BLAST (18) and performed a literature search to examine potential roles for three genes without a predicted function: the differentially expressed gene *yjjZ*, and the differentially variable genes *yoeD* and *yehS*.

- *yjjZ* aligns well (100%) with annotated membrane proteins in a variety of *Escherichia coli* strains. In *Shigella* species, there is >96% alignment with hypothetical proteins. As mentioned in the main text, computational studies suggest that *yjjZ* encodes a small RNA(19).
- *yoeD* has more than 95% alignment with transposases in *E. coli* and *Shigella* species, *Citrobacter rodentium,* and *Proteus vulgaris.*
- *yehS* has 100% alignment with hypothetical proteins in various *E. coli* and *Shigella* species. Interestingly, a 436 bp portion of the gene has 78% local alignment with DNA polymerase I in *Klebsiella oxytoca* KONIH1.

Supplementary References

1. **Erickson KE**, **Otoupal PB**, **Chatterjee A**. 2015. Gene Expression Variability Underlies Adaptive Resistance in Phenotypically Heterogeneous Bacterial Populations. ACS Infect Dis **1**:555–567.
2. **Trapnell C**, **Roberts A**, **Goff L**, **Pertea G**, **Kim D**, **Kelley DR**, **Pimentel H**, **Salzberg SL**, **Rinn JL**, **Pachter L**. 2012. Differential gene and transcript expression analysis of RNA-seq experiments with TopHat and Cufflinks. Nat Protoc **7**:562–78.
3. **Anders S**, **Huber W**. 2010. Differential expression analysis for sequence count data. Genome Biol **11**:R106.
4. **Benjamini Y**, **Hochberg Y**. 1995. Controlling the False Discovery Rate: A Practical and Powerful Approach to Multiple Testing. J R Stat Soc **57**:289–300.
5. **Langmead B**, **Salzberg SL**. 2012. Fast gapped-read alignment with Bowtie 2. Nat Methods **9**:357–9.
6. **Li H**, **Handsaker B**, **Wysoker A**, **Fennell T**, **Ruan J**, **Homer N**, **Marth G**, **Abecasis G**, **Durbin R**. 2009. The Sequence Alignment/Map format and SAMtools. Bioinformatics **25**:2078–9.
7. **Mckenna A**, **Hanna M**, **Banks E**, **Sivachenko A**, **Cibulskis K**, **Kernytsky A**, **Garimella K**, **Altshuler D**, **Gabriel S**, **Daly M**, **DePristo MA**. 2010. The Genome Analysis Toolkit: a MapReduce framework for analyzing next-generation DNA sequencing data. Genome Res **20**:1297–303.
8. **Thorvaldsdóttir H**, **Robinson JT**, **Mesirov JP**. 2013. Integrative Genomics Viewer (IGV): high-performance genomics data visualization and exploration. Brief Bioinform **14**:178–92.
9. **Huang DW**, **Sherman BT**, **Tan Q**, **Collins JR**, **Alvord WG**, **Roayaei J**, **Stephens R**, **Baseler MW**, **Lane HC**, **Lempicki R a**. 2007. The DAVID Gene Functional Classification Tool: a novel biological module-centric algorithm to functionally analyze large gene lists. Genome Biol **8**:R183.
10. **Keseler IM**, **Collado-Vides J**, **Santos-Zavaleta A**, **Peralta-Gil M**, **Gama-Castro S**, **Muñiz-Rascado L**, **Bonavides-Martinez C**, **Paley S**, **Krummenacker M**, **Altman T**. 2011. EcoCyc: a comprehensive database of Escherichia coli biology. Nucleic Acids Res **39**:D583–D590.
11. **Qi LS**, **Larson MH**, **Gilbert L a**, **Doudna J a**, **Weissman JS**, **Arkin AP**, **Lim W a**. 2013. Repurposing CRISPR as an RNA-guided platform for sequence-specific control of gene expression. Cell **152**:1173–83.
12. **Livak KJ**, **Schmittgen TD**. 2001. Analysis of Relative Gene Expression Data Using Real-Time Quantitative PCR and the 2< sup>− ΔΔCT</sup> Method. methods **25**:402–408.
13. **Luria SE**, **Delbruck M**. 1943. Mutations of bacteria from virus sensitivity to virus resistance. Genetics **28**:491–511.

14. **Sarkar S**, **Ma WT**, **Sandri GH**. 1992. On fluctuation analysis: a new, simple and efficient method for computing the expected number of mutants. Genetica **85**:173–179.

15. **Hall BM**, **Ma C-X**, **Liang P**, **Singh KK**. 2009. Fluctuation analysis CalculatOR: a web tool for the determination of mutation rate using Luria-Delbruck fluctuation analysis. Bioinformatics **25**:1564–5.

16. **Baba T**, **Ara T**, **Hasegawa M**, **Takai Y**, **Okumura Y**, **Baba M**, **Datsenko K a**, **Tomita M**, **Wanner BL**, **Mori H**. 2006. Construction of Escherichia coli K-12 in-frame, single-gene knockout mutants: the Keio collection. Mol Syst Biol **2**:2006.0008.

17. **Lamprecht MR**, **Sabatini DM**, **Carpenter AE**. 2007. CellProfiler: Free, versatile software for automated biological image analysis. Biotechniques **42**:71–75.

18. **Zheng Zhang, Scott Schwartz, Lukas Wagner and WM**. 2000. A Greedy Algorithm for Aligning DNA Sequences. J Comput Biol **7**:203–214.

19. **Chen S**, **Lesnik EA**, **Hall TA**, **Sampath R**, **Griffey RH**, **Ecker DJ**, **Blyn LB**. 2002. A bioinformatics based approach to discover small RNA genes in the Escherichia coli genome. BioSystems **65**:157–177.
