# Supplementary figures and images for: Transcriptome-Level Signatures in Gene Expression and Gene Expression Variability during Bacterial Adaptive Evolution
Source: mSphere. 2017 Feb 15;2(1):e00009-17. doi: 10.1128/mSphere.00009-17 (PMC5311112; doi:10.1128/mSphere.00009-17)

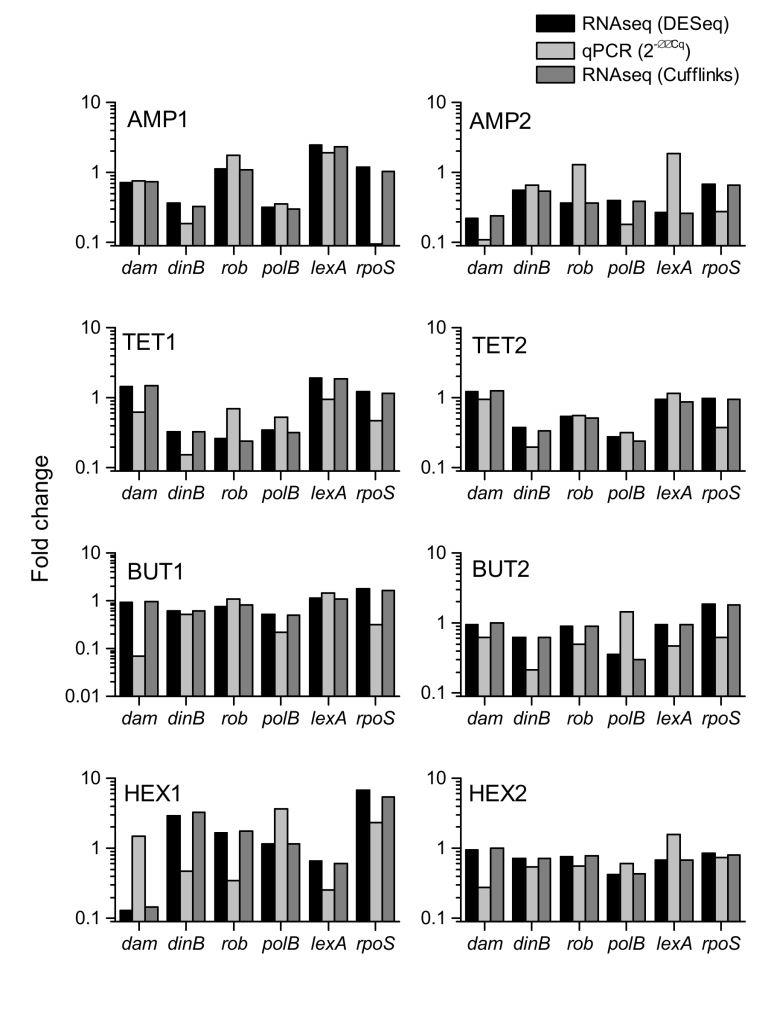

Supplement: FIG S2 [file sph001162232sf3.tif]

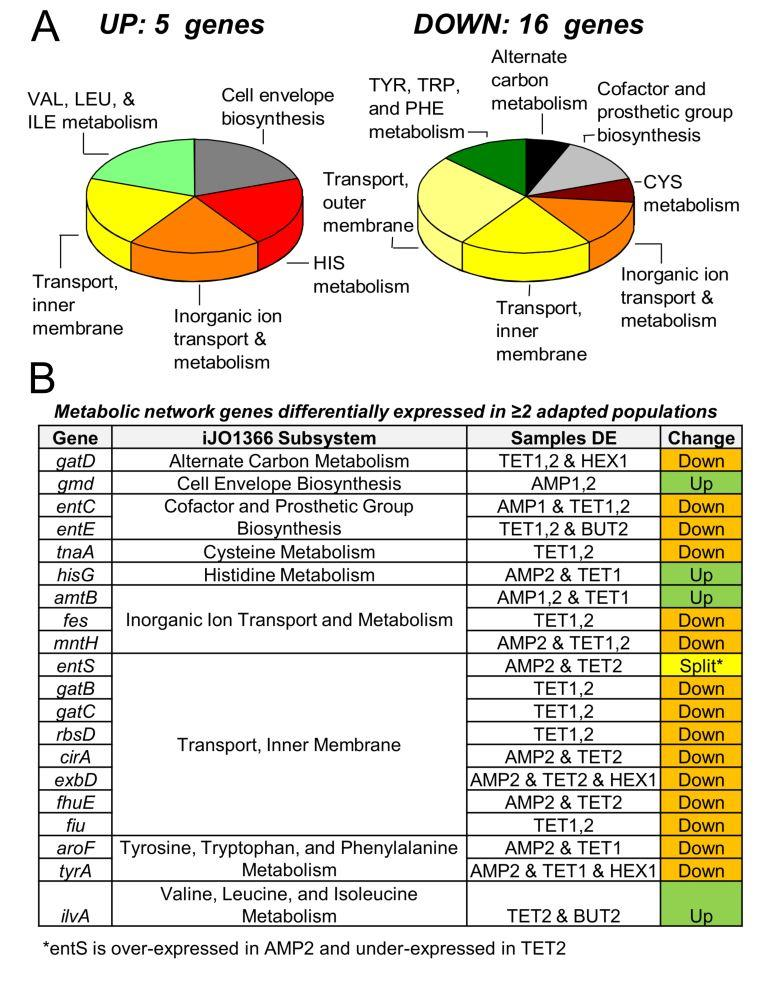

Supplement: FIG S3 [file sph001162232sf4.tif]

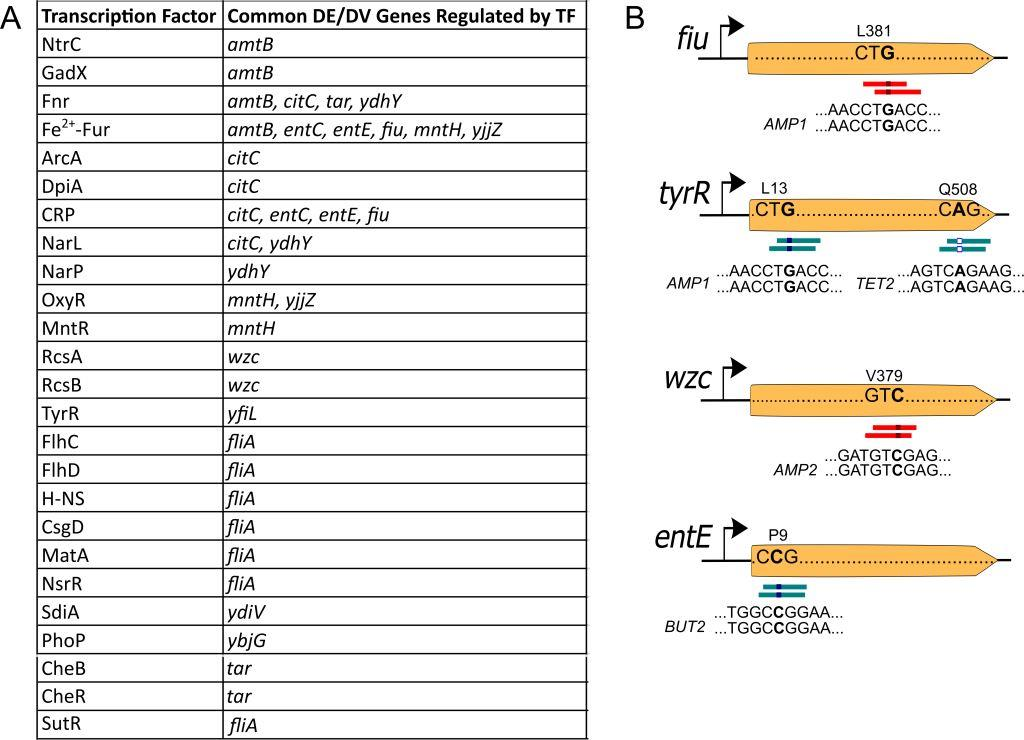

Supplement: FIG S4 [file sph001162232sf5.tif]
