# Supplementary material for: Transcriptome-Level Signatures in Gene Expression and Gene Expression Variability during Bacterial Adaptive Evolution
Source: mSphere. 2017 Feb 15;2(1):e00009-17. doi: 10.1128/mSphere.00009-17 (PMC5311112; doi:10.1128/mSphere.00009-17)
Supplement: TABLE S1 [file sph001162232st8.docx]

**Table S1:** Plasmids used in the study

| **Name** | **Purpose** | **Source** |
| --- | --- | --- |
| pdCas9 | Expresses dCas9 under aTc induction on a medium copy chloramphenicol resistance vector | Addgene Plasmid 44249 (Qi et al. 2013) |
| pgRNA-bacteria | Constitutively expresses sgRNA targeting the coding sequence of RFP on a high copy ampicillin resistance vector | Addgene Plasmid 44251 (Qi et al. 2013) |
| pRFP-i | Combines pdCas9 and pgRNA-bacteria into one medium copy chloramphenicol resistance vector | This Study |
| pyjjZ-i | Modified pRFP-i inhibiting expression of *yjjZ* | This Study |
| pwzc-i | Modified pRFP-i inhibiting expression of *wzc* | This Study |
| ptar-i | Modified pRFP-i inhibiting expression of *tar* | This Study |
| pfiu-i | Modified pRFP-i inhibiting expression of *fiu* | This Study |
| pfliA-i | Modified pRFP-i inhibiting expression of *fliA* | This Study |
| pybjG-i | Modified pRFP-i inhibiting expression of *ybjG* | This Study |
| pyoeD-i | Modified pRFP-i inhibiting expression of *yoeD* | This Study |
| pydiV-i | Modified pRFP-i inhibiting expression of *ydiV* | This Study |
| pyehS-i | Modified pRFP-i inhibiting expression of *yehS* | This Study |
