# Supplementary material for: Transcriptome-Level Signatures in Gene Expression and Gene Expression Variability during Bacterial Adaptive Evolution
Source: mSphere. 2017 Feb 15;2(1):e00009-17. doi: 10.1128/mSphere.00009-17 (PMC5311112; doi:10.1128/mSphere.00009-17)
Supplement: TABLE S2 [file sph001162232st9.docx]

**Table S2:** Primers for qPCR, Sanger sequencing, and cloning

| **Name** | **Sequence (5' - 3')** | **Purpose** |
| --- | --- | --- |
| *rrsA forward* | AACACATGCAAGTCGAACGG | qPCR |
| *rrsA reverse* | AATCCCATCTGGGCACATCC |  |
| *gyrA forward* | GTCATAGACCGCCGAGTCAC |  |
| *gyrA reverse* | GCGATGTCGGTCATTGTTGG |  |
| *fiu forward* | CGATGAGGAACCATCCTGGG |  |
| *fiu reverse* | GTAAGAAACGCGTCGAAGGC |  |
| *fliA forward* | TCCAGTTGCCCTATTGCCTG |  |
| *fliA reverse* | ACGCTATGACGCCCTACAAG |  |
| *tar forward* | AGGTTAATGCGCGTTTGCAG |  |
| *tar reverse* | AATGGTGCTGGGGGTATTCG |  |
| *wzc forward* | TGGTCACTTTCACCGTCTCG |  |
| *wzc reverse* | GTTGATTCGTTCGCGTCTGG |  |
| *yjjZ forward* | TATCGTCAGATCAGTGGCGG |  |
| *yjjZ reverse* | TGCCAGCAATAAGTGCCAGG |  |
| *ydiV forward* | CAATGCCTCCCGCAATGATG |  |
| *ydiV reverse* | TTGGGAGCAGGCAATAGCAC |  |
| *ybjG forward* | CAAGATAAACGCGCGACCAG |  |
| *ybjG reverse* | TCATTCCCAAGCGATCACGG |  |
| *yehS forward* | CCGGAGCAGACTCATCCTTG |  |
| *yehS reverse* | TGGGTAATGTCGAAGCCACC |  |
| *ydhY forward* | TTACGGTGGCCATCATCCAC |  |
| *ydhY reverse* | CCTGCCGTCAATGCAAAGAG |  |
| *yoeD forward* | TTCCATGGCAGGAGTTCGTC |  |
| *yoeD reverse* | CCTGCCGTCTGAACGGTATC |  |
| *sgRNA reverse* | GGGCCCGGGCCCAAGCTTCAAAAAAAGCACCG | Cloning and sequencing for CRISPRi |
| *fiu-i forward* | AAACCTAGGTATAATACTAGTgtccctttaacgctaacaaaGTTTTAGAG |  |
| *fliA-i forward* | AAACCTAGGTATAATACTAGTtcgctcacaaataggtaatgGTTTTAGAG |  |
| *tar-i forward* | AAACCTAGGTATAATACTAGTcgcggatacggttaatcataGTTTTAGAG |  |
| *wzc-i forward* | AAACCTAGGTATAATACTAGTcaacatgccgctccggtaacGTTTTAGAG |  |
| *ybjG-i forward* | AAAACTAGTtatctctctctaagtttaaaGTTTTAGAGCTAGAAATAGC |  |
| *yoeD-i forward* | TTTACTAGTtatggcaagtctgccgtccaGTTTTAGAGCTAGAAATAGC |  |
| *ydiV-i forward* | AGGACTAGT ttagtcttactatttctaagGTTTTAGAGCTAGAAATAGC |  |
| *yehS-i forward* | ATTACTAGTcgcacgcgatgtaaaactttGTTTTAGAGCTAGAAATAGC |  |
| *CRISPR sequencing* | aaataggcgtatcacgaggc |  |
| *fiu sequencing forward* | GCGTAAATTGCGAAGGTATC | Sanger sequencing to analyze variant calls. Forward and reverse primers used for colony PCR, just forward primer used for sequencing of the PCR product |
| *fiu sequencing reverse* | CACGACATCAACGATAACAC |  |
| *tyrR sequencing forward (Amp 1)* | ATGCAATATCGGGTGCTGAC |  |
| *tyrR sequencing reverse (Amp 1)* | CAGATGCTCACGTTCGGAAG |  |
| *tyrR sequencing forward (Tet 2)* | CCACAGGATATTTTGTTGCC |  |
| *tyrR sequencing reverse (Tet 2)* | TGCCGTTGTGGTTATTGAC |  |
| *entE sequencing forward* | TGTGACAGCGAAGGTAAC |  |
| *entE sequencing reverse* | GTTTCACCAGGTTTAATGCC |  |
| *wzc sequencing forward* | TAGACGCTAATACCGTGTTC |  |
| *wzc sequencing reverse* | CAAGCTGTACACCAAAGTTC |  |
